# Recent Researches on Scarlatina

**Published:** 1887-07

**Authors:** Wm. Osler


### EDITORIAL DEPARTMENT.

### RECENT RESEARCHES ON SCARLATINA.

BY WM. OSLER, M. D.

Several very important investigations are in progress in Great Britain relating to the etiology of this disease. Klein, of London, has attempted to prove a connection between a certain disease of the cow and scarlatina. Mr. Blyth traced an epidemic to the milk coming from a special dairy farm at Hendon, and on investigation it was found that some of the cows had sores on the skin and ulcers on the udder and teats. As described in the British Medical Journal, "the disease is ushered in by a short initiatory fever, a dry, hacking cough, sometimes quickened breathing, sore throat in severe cases, discharges from the nostrils and eyes, an eruption on the skin around the eyes, on the hind quarters and on the teats and udder, this eruption being in the form of vesicles or bullæ." Cultivations were made for micrococcus from this disease, and a similar affection was produced in calves, with post-mortem lesions resembling those of human scarlatina. Klein has obtained a micrococcus of similar characters in the blood and tissues of the subjects of the human disease. He claims also to have found the organisms in tins of condensed milk, which were suspected in connection with an epidemic of scarlet fever. Professors Axe and Brown of the Royal Veterinary College, are investigating the subject, and we shall await their report with interest.

Meanwhile, Drs. Jamieson and Edington, of Edinburgh, have issued an elaborate report on the Contagium of Scarlet Fever and described a diplococcus and a bacillus. Inoculations of the latter causes in calves fever, with a red dry rash, followed by desquamation. It does not appear that direct inoculations of the calf with scarlatinal blood have been made to ascertain if a disease similar to the Hendon affection could be produced.

It is probable that we are on the eve of important discoveries which may enable us to recognize the cause, and so help in the prevention of this dreaded scourge.

